# Distribution of different classes of CSF3R mutations and co-mutational pattern in 360 myeloid neoplasia

**DOI:** 10.1007/s00277-025-06232-1

**Published:** 2025-02-05

**Authors:** Rossana Maffei, Ambra Paolini, Benedetta Conte, Giovanni Riva, Vincenzo Nasillo, Federica Cretì, Silvia Martinelli, Francesca Giacobbi, Giorgia Corradini, Flora Pilato, Daniela Bernabei, Cesare Lancellotti, Giulia Debbia, Monica Morselli, Leonardo Potenza, Davide Giusti, Elisabetta Colaci, Francesca Bettelli, Paola Bresciani, Angela Cuoghi, Andrea Gilioli, Andrea Messerotti, Valeria Pioli, Monica Maccaferri, Giovanna Leonardi, Rossella Manfredini, Roberto Marasca, Albino Eccher, Mario Luppi, Fabio Forghieri, Anna Candoni, Enrico Tagliafico

**Affiliations:** 1 Department of Laboratory Medicine and Pathology, Diagnostic Hematology and Clinical Genomics, Azienda Ospedaliero-Universitaria, Policlinico, and AUSL Modena, Italy; 2 Hematology Unit, Department of Medical and Surgical Sciences, University of Modena and Reggio Emilia, Azienda Ospedaliero-Universitaria, Policlinico, Modena, Italy; 3 Pathology Unit, Department of Medical and Surgical Sciences, University of Modena and Reggio Emilia, Azienda Ospedaliero-Universitaria, Policlinico, Modena, Italy; 4 Interdepartmental Centre for Stem Cells and Regenerative Medicine, University of Modena and Reggio Emilia, Modena, Italy; 5 Department of Biomedical, Metabolic and Neural Sciences, University of Modena and Reggio Emilia, Modena, Italy

**Keywords:** CSF3R, Myeloid neoplasia, Co-mutational pattern, Disease outcome

## Abstract

The colony-stimulating factor 3 receptor (CSF3R) plays an essential role in differentiation, growth, and survival of granulocytes. Driver mutations in CSF3R gene represent a diagnostic marker of chronic neutrophilic leukemia (CNL). Less commonly, these mutations are observed in other myeloid neoplasms but their pathogenetic and prognostic role is still unclear. Here, we analyzed a large cohort of myeloid neoplasms to evaluate the incidence of CSF3R mutations and co-mutational profile. Mutational analysis was performed using targeted NGS myeloid panel in a consecutive cohort of 360 patients with myeloid neoplasms. Mutations in CSF3R were identified in 20/360 (5.6%) cases. A CSF3R gene mutation was present in 13/179 AML cases (7.3%), in 2/27 (7.4%) CMML cases, in 1/94 (1.1%) MDS cases and in 4/60 (6.7%) other myeloid neoplasms. The frequencies of patients with CSF3R mutations lowered to 2.8% in all cases and 3.4% in AML, excluding cases with variants of uncertain significance (VUS). A total of 23 mutations of CSF3R gene were detected, half localized in the extracellular domain, 5 in the transmembrane region (type I) and 6 mutations in the cytoplasmic domain (type II). In AML, CSF3R mutations were more frequent in patients harboring CBF alterations (25.0%) and CEBPA mutations (11.8%). Two cases with AML harboring pathogenic CSF3R variants were primary refractory to induction therapy. CMML cases with T618I variant showed a myeloproliferative phenotype. Overall, our findings support the notion that CSF3R variants, particularly type I and II pathogenic mutations, may modulate the phenotypic features of leukemic cells in myeloid neoplasia.

**Supplementary Information:**

The online version contains supplementary material available at 10.1007/s00277-025-06232-1.

## Introduction

The colony-stimulating factor 3 receptor (CSF3R), also known as granulocyte colony-stimulating factor (G-CSF) receptor, is a member of the hematopoietin receptor superfamily and plays an essential role in driving the differentiation, growth and survival of granulocytes, in particular neutrophils [1–3]. Through the binding to the G-CSF, the receptor sustains conformational changes and triggers the activation of multiple signaling pathways as for instance RAS–MAPK, PI3K-AKT and JAK-STAT pathways [4, 5].

Driver mutations in CSF3R gene represent a specific diagnostic hallmark of chronic neutrophilic leukemia (CNL), a myeloproliferative neoplasm (MPN) defined by neutrophilia, hypercellular bone marrow and poor prognosis [6, 7]. The frequency of CSF3R somatic mutations is reported to be of 80% in patients with CNL and, less commonly, these mutations are observed in atypical chronic myeloid leukemia (aCML, 40%), severe congenital neutropenia (SCN, 30%), chronic myelomonocytic leukemia (CMML, 5%) and acute myeloid leukemia (1.7% adult AML and 1.9% pediatric AML) [8–11].

CSF3R mutations fall in 3 main classes: membrane proximal point mutations, cytoplasmatic domain truncating mutations and extracellular domain mutations. First class alterations are point mutations affecting transmembrane proximal domain that determine CSF3R constitutive activation and ligand-independent growth. In particular, the CSF3R T618I is the most recurrent point mutation detected in CNL and aCML but occasionally even in CMML and AML [12, 13]. Second class mutations are frameshift or nonsense mutations typically arising in CNL, resulting in the receptor overexpression and increased sensitivity to its ligand. The third class are missense or truncating mutations affecting the extracellular domain of the receptor and are usually seen in SCN. These mutations exert a dominant negative function on wild-type receptor, affecting ligand-binding and deteriorating the neutrophil production.

Somatic CSF3R mutations are uncommon in AML and are associated with additional genetic alterations in transcription factors including double mutated CCAAT/enhancer binding protein α (CEBPA^dm^), core-binding factors (RUNX1-RUNX1T1 and CBFB-MYH11) and rarely NPM1 [10, 14–16]. In line with the two-hit model of leukemogenesis, the evolution of a leukemia phenotype requires step-wise acquisition of genetic alterations, inducing block of differentiation and proliferative advantage [17]. According to this, CSF3R mutation is not sufficient to sustain leukemogenesis but additional mutations are required, as demonstrated in mouse model by the group of Beekman et al. [18]. Genetic aberrations mediating a blockade of differentiation, such as CEBPA mutations or CBF fusions, must occur prior to CSF3R mutations to promote AML development. In a later stage, CSF3R mutations synergistically participate to drive the development of immature myeloid cells in AML thus providing a proliferative advantage [19–21]. Of note, a recent study demonstrated that CSF3R (T618I) synergizes with CEBPA^dm^ to induce AML in mouse models [20].

The prognostic significance of CSF3R mutations in myeloid neoplasia is not well defined. Significant lower relapse-free survival (RFS) and overall survival (OS) were observed in CEBPA^dm^ AML patients with concomitant mutated CSF3R gene [22, 23]. Conversely, AML patients with RUNX1-RUNX1T1 and CSF3R mutations showed comparable clinical outcome [15]. If CSF3R mutations, rarely observed in NPM1 mutated AML (0.9%), confer poor prognosis remains unclear [24]. Mutated CSF3R can activate SRC-family tyrosine kinases (TNK2), MAPK and JAK-STAT signaling pathways. Thus, several studies confirmed that JAK and MEK1/2 inhibition could induce a therapeutical response in patients with these mutations [25, 26].

In the current study, we performed mutational analysis using targeted next-generation sequencing (NGS) in a consecutive cohort of 360 patients diagnosed with myeloid neoplasms, to identify mutations in CSF3R gene in different subgroups. We characterized patients harboring CSF3R mutated gene throughout a molecular, morphologic and immunophenotypic analysis to define the frequency, co-mutational profile and characteristics of these patients.

## Patients and methods

### Patients

We analyzed 360 patients with myeloid neoplasms (179 AML, 94 MDS, 60 MPN, 27 CMML) diagnosed between January 2020 and May 2024, at our Hematology Unit at Modena University Hospital. The hematological and clinical features are shown in supplementary Tables. Type and schedule of treatment, as well as the response to treatment, timing and treatment of relapse, and the date of the last follow up and the survival status, were also reported.

### Next-generation sequencing (NGS)

Targeted sequencing (minimal read depth at 500X) was performed on 30 genes involved in hematologic disorders. DNA was extracted from peripheral blood (PB) or bone marrow (BM) using the Maxwell^®^ 16 LEV Blood DNA Kit and then quantified through the Qubit^®^ dsDNA HS Kit (Thermo Fisher Scientific, USA). The sequencing was then performed using the Myeloid Solutions™ Panel (MYS) (SOPHiA Genetics, Saint Sulpice, Switzerland), a hybridization capture-based solution covering exonic and intronic regions of 30 genes frequently involved with myeloid neoplasms. Libraries were prepared starting from 200 ng of gDNA according to the manufacturer’s instructions, quantified with Qubit HS Kit and analyzed with Bioanalyzer HS DNA Kit (Agilent, USA). Briefly, DNA went through a first step of enzymatic fragmentation, end repair and A-tailing. DNA fragments were then ligated to dual-barcoded adapters followed by a phase of dual-size selection. Libraries were amplified, cleaned up, quantified through the Qubit and finally pooled and lyophilized. Targets were then hybridized with SG probes and after steps of clean up, a post-capture amplification was performed followed by the quantification with the Qubit and the quality and size assessment using the Bioanalyzer. Paired-end sequencing was ultimately performed using the MiSeq Reagent Kit V3 on the Illumina MiSeq instrument with 24 samples per run. The panel has a threshold of 2% for low-frequency variants and the sequence alignment, base calling and variant annotation for CNVs and SNVs were performed using SOPHiA DDM platform. The identified variants are then classified in five groups according to ACMG [27] and AMP/ASCO/CAP [28]. Varsome (https://varsome.com/) and Franklin (https://franklin.genoox.com/) tools were used to further explore the evidence for classification of pathogenicity. Likely benign or benign variants were excluded from the analysis.

### Morphologic and histological assessment

Wright–Giemsa stained PB and BM aspirate smears, and hematoxylin-eosin stained core biopsy specimens were reviewed. The tissue samples were evaluated in conjunction with immunohistochemistry (IHC) stains. The PB and BM smears and tissue sections were assessed for cellularity, blast percentage, and morphologic dysplasia. BM myelofibrosis (MF) was evaluated by reticulin and trichrome stains performed on the BM core biopsy specimens when available. The grade of MF was based on the criteria of the European Consensus on the grading of BM fibrosis [29, 30].

## Results

### Distribution and type of CSF3R mutations in myeloid neoplasia

**Fig. 1:**
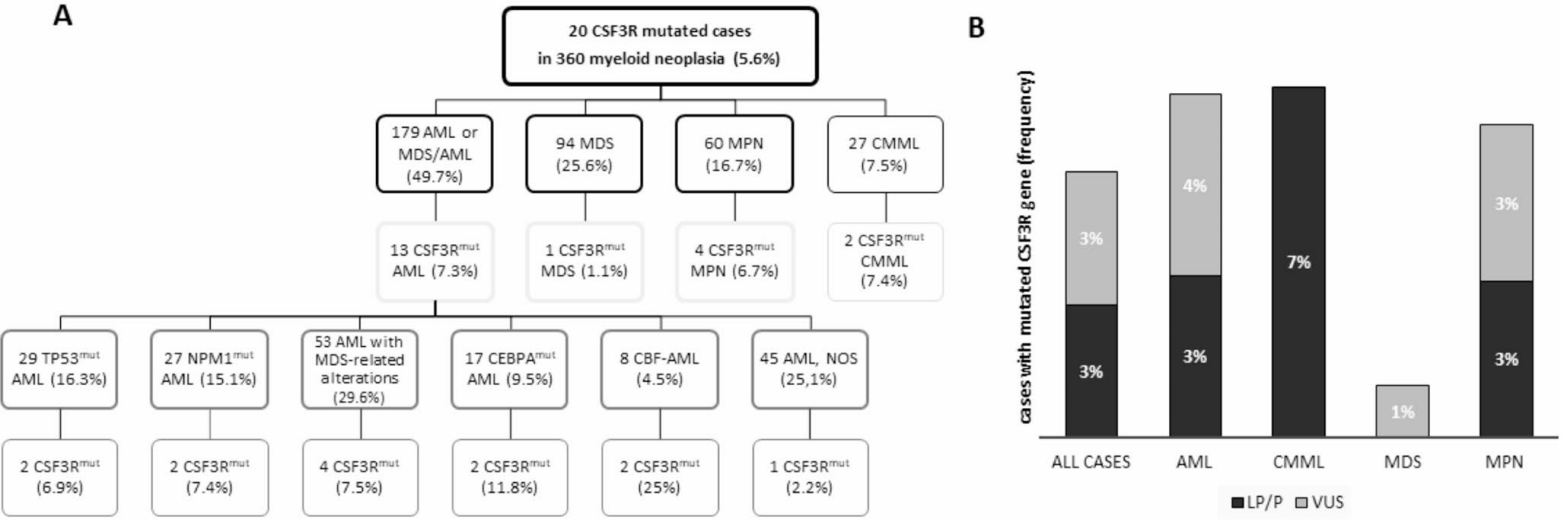
Frequencies of CSF3R mutated cases in a cohort of 360 patients with myeloid neoplasia. (**A**) Flow-chart shows the distribution of CSF3R mutated cases among patients with AML or MDS/AML, MDS, MPN or CMML. For 179 AML cases frequencies are reported, as percentage compared to all AML cases, among subsets i.e., TP53-mutated, NPM1-mutated, CEBPA-mutated, AML with CBL alterations, AML with MDS-related mutations. (**B**) Histograms show frequencies of cases harboring pathogenetic (P) or likely pathogenetic (LP) variants or variants of uncertain significance (VUS) among different myeloid neoplasia

**Fig. 2:**
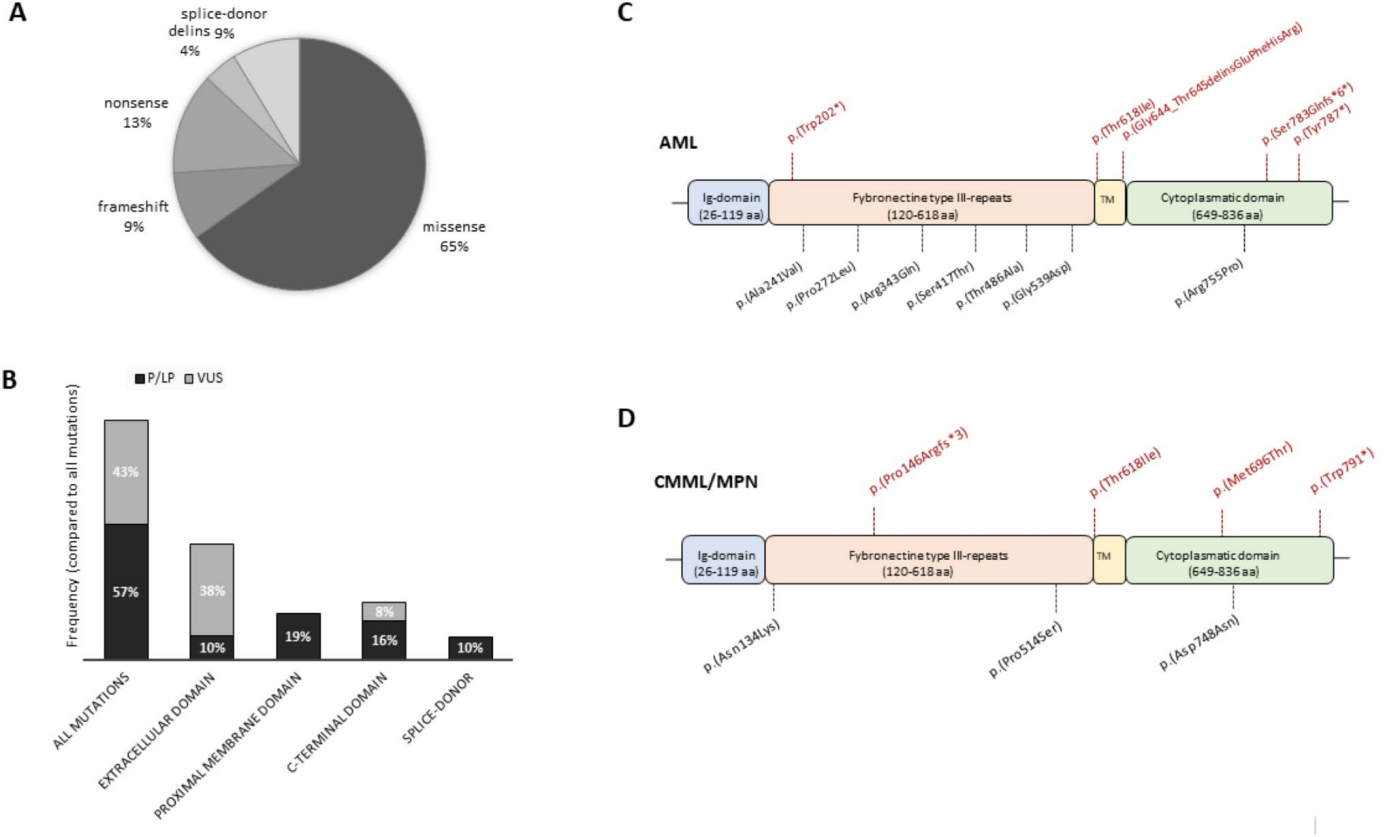
Characteristics and distribution of CSF3R mutations in patients with myeloid neoplasia. (A) Pie-chart depicts the frequencies of different types of CSF3R mutations i.e., missense, frameshift, nonsense, delins and splice-donor variants. (B) Distribution of pathogenic/likely pathogenic (P/LP) mutations and variants of uncertain significance (VUS) in extracellular, proximal membrane, C-terminal domains of CSF3R gene or splice-donor mutations. (C) Pictures show the different domains of CSF3R gene and distribution of mutations in AML (above) and CMML (below). In red, pathogenic/likely pathogenic (P/LP) mutationsand in black, variants of unknown significance (VUS).

Mutations in CSF3R were identified in 20 (5.6%) of 360 patients with myeloid neoplasia (Fig. 1A). Among AML or MDS/AML patients (*n* = 179), CSF3R-mutated gene was present in 13 cases (7.3%). One patient with CSF3R mutations was observed in MDS, 2 cases in CMML and 4 in other myeloproliferative neoplasia (1 CNL, 1 hypereosinophilia, 1 secondary myelofibrosis MF post-PV and 1 primary MF), accounting for frequencies of 1.1%, 7.4% and 6.7% respectively (Fig. 1). A total of 23 mutations of CSF3R gene were detected (Table 1 and Supplementary Tables 1 and 2). Three patients showed 2 concomitant CSF3R mutations. Thirteen CSF3R mutations (57%) were classified as pathogenic (P) or likely pathogenic (LP), the remaining variants were defined as of uncertain significance (VUS). Considering exclusively pathogenetic or likely pathogenetic variants, the frequency of patients with CSF3R mutations lowered to 2.8% in all cases and 3.4% in AML (Fig. 1B). The majority of CSF3R mutations were missense (15/23, 65.2%), 3 nonsense (13.0%), 2 splice-donor (8.7%), 2 frameshift (8.7%) and 1 delins inframe mutation (4.3%) (Fig. 2A). Half of mutations were localized in the extracellular domain, 5 in the transmembrane or proximal membrane region (type I mutations) [4 T618I in exon 14 and one p.(Gly644_Thr645delinsGluPheHisArg) in exon 15] and 6 mutations in the cytoplasmic domain (type II mutations) (Fig. 2B). Pathogenic variants were more frequent in the proximal membrane region and in the cytoplasmic tail, whereas the majority of CSF3R mutations in the extracellular domain were missense variants of uncertain significance.

**Table 1:** CSF3R variants found in patients with myeloid neoplasia

Patient	sex-age	Diagnosis	Karyotype	Variant (nucleotide)	Variant (protein)	Mutation type	Exon	VAF (%)	fathmm-MKL score ^1^	fathmm-MKL ^1^	MutationTaster score^2^	MutationTaster ^2^	DANN Score^3^	Pathogenicity	Localization	co-mutations (VAF%; pathogenicity)/fusions
1	F-41	AML	-7	c.2346dup; c.1853 C > T	p.(Ser783Glnfs*6); p.(Thr618Ile)	frameshift missense	17; 14	41.8; 44.0	-; 0.9578	-;Damaging	-; 0.6817	-; Deleterious	-;0.9984	P; P	cytoplasmic tail; transmembrane proximal membrane	DNMT3A (47.8;LP); DNMT3A (46.8;LP)
2	M-57	AML	normal	c.2361T > G	p.(Tyr787*)	nonsense	17	38.9	0.8194	Damaging	0.9852	Deleterious	0.9941	LP	cytoplasmic tail	DNMT3A(43.4; P); NPM1 (39.5; P)
3	M-46	AML	t(8;21)	c.1474 + 1G > A	p.(?)	splice_donor_+1	17	22.5	0.9715	Damaging	1	-	0.9893	LP	splicing factor	EZH2 (44.6; VUS)/RUNX1-RUNX1T1
4	M-70	AML	t(8;21)	c.1931_1935delinsAGTTCCACAGA	p.(Gly644_Thr645delinsGluPheHisArg)	inframe_6	15	12.8	-	-	-	-	-	LP	transmembrane proximal membrane	ASXL1 (28.9; P); KIT (10.5; P); NRAS (9.9;P)/RUNX1-RUNX1T1
5	M-78	AML relapsed	normal	c.1853 C > T	p.(Thr618Ile)	missense	14	38.6	0.9578	Damaging	0.6817	Deleterious	0.9984	P	transmembrane proximal membrane	ZRSR2 (80.2; LP); CEBPA (79.0; P); DNMT3A (41.1; LP); TET2 (40.7; LP/36.9. LP)
6	M-81	MDS/AML	-17; -Y	c.606G > A	p.(Trp202*)	nonsense	6	6.84	0.8952	Damaging	1	Deleterious	0.9965	LP	extracellular domain	DNMT3A (22.2; P); SF3B1 (6.5; P); ASXL1(4.5; P)
7	M-69	AML	normal	c.1028G > A	p.(Arg343Gln)	missense	9	49.5	0.1953	Benign	0.9986	Benign	0.9723	VUS	extracellular domain	DNMT3A (86.3; LP); TET2 (45.4;LP); PTPN11 (45.2; P); TET2 (43.4;LP)
8	F-72	AML	+ 8; del(13q14)	c.1249T > A	p.(Ser417Thr)	missense	10	49.5	0.8899	Damaging	0.9898	Benign	0.9931	VUS	extracellular domain	TET2 (67.1; LP); TP53 (46.5; P); CALR (39.6; P); NRAS (28.9; P)
9	F-29	AML	+ 8;+mar (12)	c.1616G > A	p.(Gly539Asp)	missense	13	47.5	0.8765	Damaging	0.5582	Benign	0.9947	VUS	extracellular domain	FLT3 (9; P); NPM1 (8.6; P)
10	M-75	AML	normal	c.815 C > T	p.(Pro272Leu)	missense	7	50.5	0.9149	Damaging	0.9999	Benign	0.9984	VUS	extracellular domain	IDH2 (50.1;P); SRSF2 (48.4;P); ASXL1 (44.7;P)
11	M-82	AML	complex	c.1456 A > G	p.(Thr486Ala)	missense	11	46.5	0.07165	Benign	1	Benign	0.8606	VUS	extracellular domain	TP53 (37.6; P)
12	F-81	AML	-7	c.722 C > T	p.(Ala241Val)	missense	7	48.7	0.00975	Benign	1	Benign	0.6054	VUS	extracellular domain	IDH2 (47.3;P); SRSF2 (47.2;P); DNMT3A (47.0;P); CEBPA (6.1;P); RUNX1 (47.0;P)
13	M-22	AML	complex	c.2264G > C	p.(Arg755Pro)	missense	18	49	0.001359	Benign	1	Benign	0.9166	VUS	cytoplasmic tail	none
14	M-77	CMML	normal	c.1853 C > T	p.(Thr618Ile)	missense	14	12.1	0.9578	Damaging	0.6817	Deleterious	0.9984	P	transmembrane proximal membrane	DNMT3A (48.0; P); SRSF2 (47.3;P); RUNX1 (46.3; P); SF3B1(46.2;P)
15	M-81	CMML	-Y; mar (3)	c.437_438del c.2087T > C	p.(Pro146Argfs*3); p.(Met696Thr)	frameshift; missense	5; 17	49.7;49.7	-; 0.0282	-; Benign	-; 0.2419	-; Deleterious	-; 0.2419	LP; LP	extracellular domain; cytoplasmic tail	none
16	F-73	CNL	normal	c.2372G > A; c.1853 C > T	p.(Trp791*); p.(Thr618Ile)	nonsense; missense	17; 14	41.3; 41.0	0.9627; 0.9578	Damaging; Damaging	1; 0.6817	Deleterious;Deleterious	0.9898; 0.9984	P; P	cytoplasmic tail; transmembrane proximal membrane	ASXL1 (19.9;P)
17	M-24	Hypereosinophilia	normal	c.1474 + 1G > C	p.(?)	splice_donor_+1	-	49.2	0.9747	Damaging	1	-	0.9897	LP	splicing factor	none
18	M-60	Post-PV MF	normal	c.2242G > A	p.(Asp748Asn)	missense	17	49.9	0.1801	Benign	1	Benign	0.7072	VUS	cytoplasmic tail	MPL (45.4;P); TET2 (42.9;LP)
19	M-67	MF	normal	c.402 C > A	p.(Asn134Lys)	missense	5	46.4	0.9065	Damaging	0.9143	Benign	0.9956	VUS	extracellular domain	JAK2 (46.0;P); SF3B1 (44.7;P); SRSF2 (48.5;P)
20	M-69	MDS	normal	c.1540 C > T	p.(Pro514Ser)	missense	12	48.2	0.5924	Neutral	0.9999	Benign	0.5314	VUS	extracellular domain	NRAS (7.6;P); TET2 (13.1;P)

^1^FATHMM-MKL is a software used for the prediction of proteins coding and noncoding effects by integrating functional annotation information from the ENCODE. Range 0 to 1

^2^MutationTaster is an in-silico prediction tool able to assess the pathogenicity of a variant through the Bayer classifier. The higher the score, the more likely the variant is deleterious

^3^DANN is an annotation method based on deep neural network which gives in output a score ranging from 0 to 1. Higher values denote an increased probability to be damaging

In AML patients we found 14 CSF3R variants; among them, 7 (50.0%) were localized in the extracellular domain (one nonsense and 6 missense), 3 mutations (21.4%) in the transmembrane proximal domain (two missense T618I and one delins), 3 (21.4%) in cytoplasmatic tail (W787* nonsense variant, S783Qfs frameshift variant and a missense variant) and 1 (7.1%) is a splicing variant (Fig. 2C). In a patient with AML (AML#1), we observed two concomitant CSF3R variants, a frameshift variant in the cytoplasmic domain and the hotspot mutation T618I in the proximal membrane region, indicating that different classes of mutations may co-exist in the same patient.

Rarely observed in MDS (1.1%), CSF3R mutations were more frequent in MDS/MPN, particularly in CMML, being observed in 2/27 cases (7.4%) (Table 1). In these cases, all variants were pathogenic or likely-pathogenic, comprising T618I in one case and two variants (P146fs and M696T) in the other case (Fig. 2D). Overall, we showed that functionally pathogenic CSF3R mutations can be detected in about 3% of patients, mainly comprising AML and CMML cases.

### Co-mutational pattern of CSF3R-mutated acute myeloid leukemia

In AML, CSF3R mutations were more frequent in patients harboring core-binding factor (CBF) alterations (25%) and CEBPA mutations (11.8%), followed by AML with MDS-related alterations (mutations or cytogenetic abnormalities) (7.5%), NPM1 mutated AML (7.4%) and AML harboring TP53 mutations and/or 17p deletion (6.9%) (Table 1 and Supplementary Table 1). CBF alterations were detected in 8 AML, including 5 cases with RUNX1-RUNX1L1 fusion and 3 with CBFβ-MYH11. Uncommon CSF3R mutations were found in 2 cases, both with RUNX1-RUNX1L1. One case (AML#3) showed one splice-donor CSF3R variant and the other (AML#4) had a delins variant in the transmembrane region of CSF3R. Of AML patients with CEBPA mutations (*n* = 17), 12 cases had variants outside the bZIP domain (70.6%, CEBPAmut^outbZIP^), whereas the remaining cases showed bZIP mutations (29.4%; 3 missense mutations, CEBPAmut^bZIPmis^;1 frameshift, CEBPAmut^bZIPfs^; 1 inframe, CEBPAmut^bZIPinfr^). Among CEBPA mutated AML, 2 patients were mutated in CSF3R gene. One patient (AML#5) acquired the pathogenic variant T618I in the proximal membrane region of CSF3R gene at relapse with an allele frequency of 38.6%. The other patient (AML#12) had a missense mutation of uncertain significance (VUS) in the fibronectin-type III domain of CSF3R gene. NPM1 mutations were present in 27/179 AML cases (15.1%), including 19 NPM1 type-A, 4 type-B, 1 type-D and 3 NPM1 non-ABD mutations. Two cases showed a concomitant mutation of CSF3R gene (7.4%). One case (AML#2) harbored a nonsense mutations in the cytoplasmatic domain [p.(Tyr787*)] of CSF3R together with NPM1-nonABD and DNMT3A mutations; the other (AML#9) had a missense variant (VUS) in the extracellular domain [p.(Gly539Asp)] of CSF3R gene, NPM1-nonABD and FLT3-TKD mutations. CSF3R mutations were detected in 2 patients harboring TP53 mutated gene (2/29, 6.9%). In both cases, variants were missense mutations of uncertain significance localized in exon 10 and 11.

**Fig. 3:**
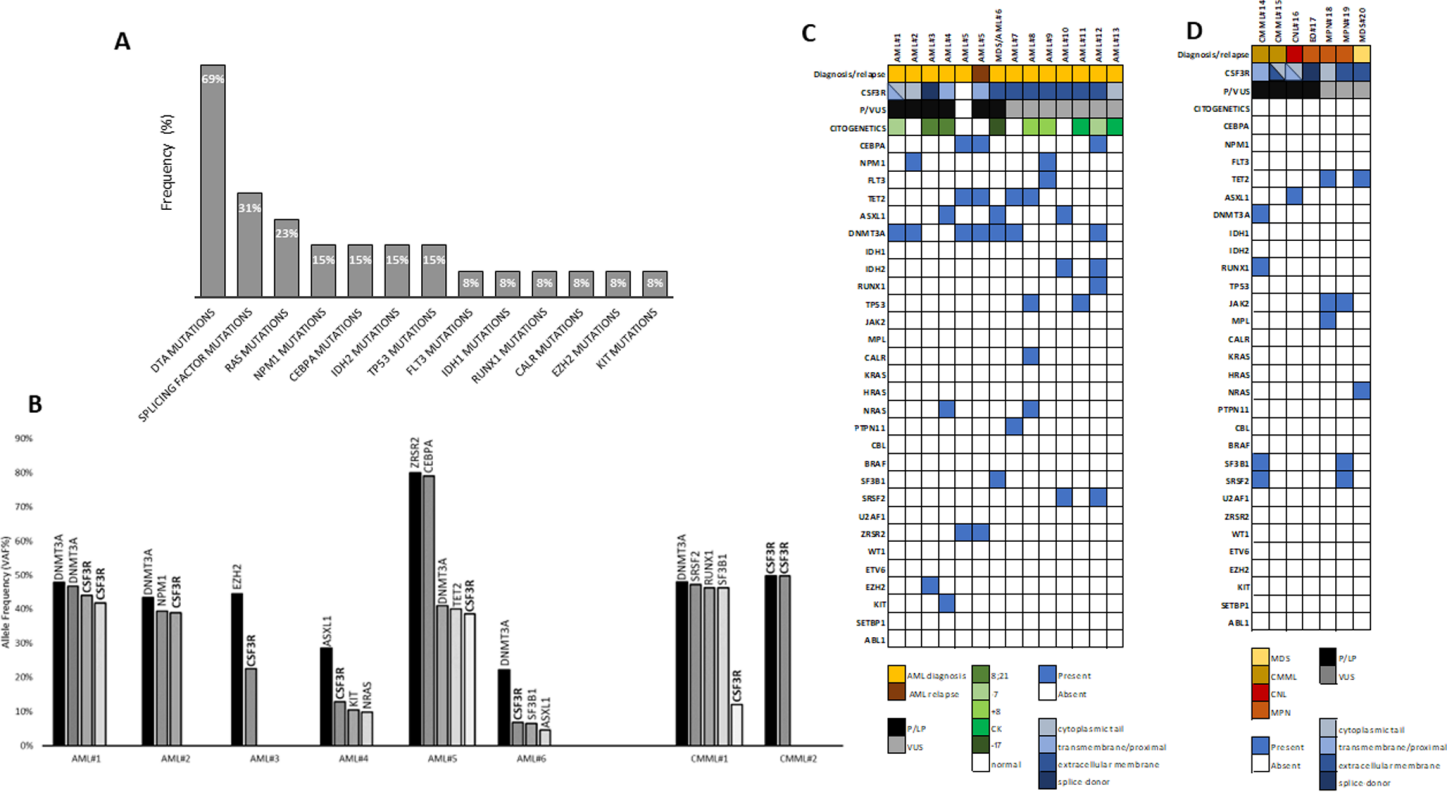
Co-mutational pattern of CSF3R-mutated cases. (**A**) Histogram show the frequencies of co-occurring mutations in other genes related to myeloid neoplasia in AML cases with CSF3R-mutated gene. (**B**) Allele frequencies (VAF%) of mutated genes in AML and CMML cases with pathogenic/likely pathogenic mutations of CSF3R gene are depicted. (**C**, **D**) Heatmaps depict co-mutational profile of CSF3R-mutated cases. Each row represents a different gene and each column an individual patient with de novo or relapsed AML (**C**) or other myeloid neoplasm (**D**). A colored cell indicates the presence of mutation, and a blank cell indicates wild-type. Color legends are depicted below

The most common co-occurring alterations in CSF3R-mutated AML patients were DTA (DNMT3A, TET2 and ASXL1) mutations (9/13, 69.2%), splicing-factor mutations (4/13, 30.8%; 1 ZRSR2, 1 SF3B1, 2 SRSF2), RAS pathway mutations (3/13, 23.1%; 2 NRAS, 1 PTPN11), CEBPA (2/13, 15.4%), NPM1 (2/13,15.4%), and TP53 (2/13, 15.4%) (Fig. 3). Multiple DTA mutations (2 or 3 concomitant variants) were present in 4 patients. In 3 out of 6 patients with AML harboring pathogenic or likely pathogenic CSF3R variants, a variant allele frequency (VAF) of 44.0%, 41.8%, 38.9% and 38.6% characterized these mutations, suggesting a dominant distribution inside the leukemic population, whereas lower VAF were detected in the other 3 cases compared to other co-occurring mutations, implying a subclonal pattern in these cases. Cytogenetic abnormalities were detected in 9/13 CSF3R-mutated AML. Two cases had the CBF translocation t(8;21) (q22;q22), two monosomy 7, one monosomy 17, three trisomy 8 and 2 had complex karyotype. Pathogenic or likely pathogenic CSF3R variants were present in 2 AML patients with normal karyotype (AML#2 and AML#5).

### Clinical outcomes of AML patients

The clinical outcome of AML patients (*n* = 6) harboring pathogenetic and VUS CSF3R variants was generally poor (Supplementary Tables 3 and 4). Two patients (2/6, 33.3%) were primary refractory: one (AML#2) received intensive chemotherapy (GIMEMA AML1819 trial; PMCID: PMC10429782), “3 + 7” plus gentuzumab ozogamicin), being diagnosed with NPM1-mutated AML and ELN favorable risk (normal cytogenetics), and the other (AML#1) received induction therapy with CPX-381, presenting with hyperleukocytosis (100k/ul), AML with cytogenetic alterations associated with MDS (monosomy 7), and ELN adverse risk. High leukocytosis at diagnosis (> 100k/µl) characterized patient with AML harboring a frameshift mutation of cytoplasmic tail. The two patients harboring t(8;21) also had poor outcome with an overall survival inferior to 1 year (Supplementary Table 3).

### CSF3R mutations in myelodysplastic and myeloproliferative neoplasia

In our cohort, CSF3R mutations were observed in 4 out of 60 patients with MPN. Two pathogenic variants localized in the proximal membrane region and in the cytoplasmic tail were present in a patient with CNL. In CSF3R-mutated patients other than AML, we also observed 2 out of 7 patients (28.6%) harboring CSF3R and two concomitant mutations of SF3B1 and SRSF2 (Table 1; Fig. 3). More frequently, pathogenic CSF3R variants can be detected in patients with CMML. In particular, we found 2 (7.4%) patients with CMML who harbored CSF3R mutations, one had CSF3R T618I missense variant (CMML#14) in exon 14 with 12.1% allele frequency and the other (CMML#15) two mutations, CSF3R M696T missense in exon 17 and CSF3R P146Rfs*3 frameshift variant in exon 5, both with 49.7% allele frequency. Patient 14 is a 77-year-old man diagnosed with CMML-type 2 and a myeloproliferative phenotype (Supplementary Table 5). A complete blood count revealed hemoglobin of 9.2 g/dl, platelet of 45 × 10^3^/µl, white blood cell count (WBC) of 25.9 × 10^3^/µl with 7 × 10^3^/µl monocytes and 20 × 10^3^/µl neutrophils. A bone marrow aspirate and biopsy were performed. Bone marrow was hypercellular with increased monocytic population, increased myeloid/erythroid ratio, 15% myeloblasts and few ring sideroblasts. Immunophenotyping analysis of peripheral blood revealed 8% myeloblasts positive for CD34, CD117, CD13, CD38 and negative for CD33. Karyotyping showed 46, XY male karyotype and NGS detected 4 co-mutations, comprising DNMT3A R736L, RUNX1 Q283* and two concomitant variants in genes coding for splicing factors, SRSF2 with the hotspot mutation P95L and SF3B1 with the hotspot mutation K666N. SRSF2 mutation is most frequent in CMML, with reports ranging from 28.4 to 47.2%, whereas mutations in SF3B1 gene are less frequent in CMML (5–10%) (Fig. 3D). However, mutations in splicing factors are reported to be infrequent in cases harboring CSF3R mutations and are generally mutually exclusive. Patient rapidly progressed to AML and died after 10 months from diagnosis (Supplementary Table 5). The other patient with CMML (#15) is an 81-year-old male with CMML-type 1. Blood count revealed hemoglobin of 12.3 g/dl, platelet of 82 × 10^3^/µl, WBC of 9.9 × 10^3^/µl with 1.38 × 10^3^/µl monocytes. Bone marrow aspirate analysis detected hypercellularity, increased myeloid/erythroid ratio, expansion of monocytic lineage (30–40%) evaluated by butyrate esterase and absence of ring sideroblasts by Perls reaction. NGS panel detected two CSF3R mutations, as sole pathogenetic variants among myeloid genes analyzed. CSF3R M696T is a missense variant in the cytoplasmic tail of CSF3R. The other variant was a likely pathogenic frameshift mutation P146Rfs in the extracellular fibronectin-like type III (FNIII) domain.

## Discussion

Here, we report a retrospective evaluation of patients diagnosed with myeloid neoplasms in our Institution since 2020, having data of mutational spectrum by an NGS myeloid gene panel. Mutations in CSF3R gene were present in 20 patients accounting for a frequency of 5.4%. However, half of CSF3R variants were classified as VUS, mainly missense mutations localized in the extracellular domain. Strictly considering cases harboring pathogenic or likely pathogenic variants, the frequency of CSF3R-mutated cases was 2.8%. Most of them (60%) were found in AML cases, mainly in the transmembrane domain and in the cytoplasmic tail of the gene or in CMML cases (20%). Our data indicate that CSF3R mutations are not exclusively present in CNL (80–90% of cases) or aCML (5–10%) but are detectable in about 3% of AML patients and 7% of CMML patients [14, 15, 18, 31, 32]. In addition, another 4% of AML showed CSF3R mutations of uncertain significance predominantly located in the extracellular domain.

Regarding the protein domains involved, we reported variants in all functional domains of CSF3R, the extracellular portion, the transmembrane and proximal membrane domain and in the cytoplasmic tail. Different functional effects characterize the different classes of variants based on the CSF3R domains involved. Mutations in the extracellular domain are loss-of-function and disrupt G-CSF binding and receptor dimerization, whereas those located in the transmembrane and C-terminal regions are activating mutations. In particular, mutations in the proximal membrane i.e., T618I, or in the transmembrane domain stabilize CSF3R dimerization in absence of the ligand and induce a constitutive activation of JAK/STAT signaling, whereas mutations in the C-terminal portion of the gene eliminate regulatory motif of signal attenuation, inducing enhanced proliferation and reduced differentiation by activation of SRC signaling cascade. It implies that the phenotypic consequences and the clinical impact of the acquisition of different classes of CSF3R mutations in myeloid cells would be extremely different.

We observed the presence of the proximal membrane mutation T618I in both AML and CMML patients. The pathogenetic impact of this variant is well established in a murine bone marrow transplantation model in which T618I is sufficient to generate a lethal myeloproliferative disorder with neutrophilic expansion sensitive to JAK1/2 inhibition [25]. T618I-transduced BaF3 showed JAK2, STAT3, STAT5, SRC, and TNK activation [12, 33]. The CSF3R T618I variant frequently co-occur with CEBPA mutations [9]. S Rohrabaugh et al. showed, using expression profiling and biochemical experiments, that an enhanced Mapk signaling is crucial to leukemogenesis by CSF3R proximal and compound mutants and that the trametinib-mediated inhibition of Mek1/2 is able to suppress leukemia induced by both CSF3R proximal and ruxolitinib-resistant mutations [26]. Of interest, CSF3R ^T618I^ mutated CEBPA^bi^ subset was uniformly sensitive to JAK inhibitors, as also reported in CSF3R T618I mutated cases in other related disorders, i.e. T acute lymphoblastic leukemia and CNL [34]. In our cohort, T618I was acquired at relapse in one case harboring biallelic mutated CEBPA gene. At relapse, we can observe the acquisition of a myeloproliferative phenotype, not present at diagnosis, with a hypercellular BM trephine biopsy reaching almost 100% cellularity, with markedly increased myeloid-erythroid ratio due to maturing neutrophilic granulopoiesis expansion and blast count about 10%. CSF3R ^T618I^ mutated CEBPA^bi^ showed a distinctive transcriptomic profile when compared to CSF3R^WT^ cases, including increased levels of genes related to myeloid maturation i.e., S100A8, S100A9, ELANE, CD117. Accordingly, CMML patient harboring CSF3R T618I mutation had a myeloproliferative phenotype with leukocytosis but showed concomitant dysplastic features, probably derived from the presence of double splicing-factor mutations, SRSF2 P95L and SF3B1 K666N. This observation is in line with previous reports on CMML, showing the predominance of myeloproliferative pattern, leukocytosis and neutrophilia in CSF3R-mutated cases. However, mutations in splicing factors are reported to be extremely rare in this subset [35–38].

Class 2 mutations were found in 3 AML cases in our cohort, 1 frameshift mutation (P), 1 nonsense (LP) and 1 missense (VUS). The patient with frameshift S783Qfs variant presented with hyperleukocytosis (100k/µl) and was primary refractory to induction therapy. Two other AML cases with this type of variant (p.L807fs and p.V777fs) were recently reported in the literature [32] and, of interest, also these patients showed leukocytosis above 100k/µl, at diagnosis. AML patients harboring NPM1 mutations were generally included in favorable risk subset accordingly to ELN stratification, when concomitantly absent high-risk cytogenetics or FLT3-ITD alteration [39–42]. Normal cytogenetics and high response rates to induction chemotherapy commonly characterize this subset. Co-occurrence of specific gene mutations in NPM1-mutated AML may modulate clinical outcome. The impact of myelodysplasia-related gene mutations, i.e. SRSF2, SF3B1, U2AF1, ZRSR2, ASXL1, EZH2, BCOR and STAG2, in this subset is currently undefined [24, 43]. Other co-mutations were reported to be associated with adverse, i.e. FLT3-ITD, DNMT3A, WT1 and TP53 gene mutations, or favorable clinical outcome, i.e. IDH1/2 and PTPN11-PTP gene [44, 45]. Pathogenic CSF3R mutations are rare in NPM1 mutated AML patients (frequency, 1–5%) and their impact on clinical behavior is unknown. In our cohort, one patient with AML harboring NPM1 mutated gene and normal karyotype unexpectedly showed primary refractoriness to induction therapy. Targeted-gene NGS identified the presence of a nonsense mutation p.Y787* located in the cytoplasmic domain of CSF3R gene, in exon 17 in one of four essential tyrosine residues in the docking sites for SH2 domain-containing proteins. This mutation may cause a loss of negative regulation with a decrease in receptor trafficking to lysosome and was previously reported in a pediatric patient with AML [46], In particular, tyrosine 787 is involved in MAPK/ERK1-2 activation pathway by recruitment of GRB2. Several truncating mutations in the cytoplasmic tail of CSF3R lead to hyper-response to G-CSF, unrestrained signaling downstream of the receptor on ligand binding, decreased receptor internalization, increased proliferation and defective differentiation. C-terminal truncation mutations were observed in SCN patients who develop myeloid neoplasia (MDS and/or AML) [47]. Accordingly, mouse model of truncated CSF3R mutations showed immature myeloid cells and mild neutropenia [48]. In a study by Zhang and colleagues two cases of AML with concomitant NPM1 and CSF3R mutated genes were reported, both harboring T618I variant and additionally in one case the missense variant Y752H in the C-terminal domain [14]. Another AML patient concomitantly mutated in NPM1 and CSF3R genes was reported in another recent study and underwent hematopoietic stem cell transplant [32]. Few reports are available to robustly assess the prognostic impact of these mutations. However, it might be supposed that CSF3R type II mutations may contribute to enhanced proliferation and blast accumulation due to activated SRC downstream signaling. Recently, a study identified the minimal truncated elements necessary for CSF3R leukemogenic potential, defining that truncation mutations between T738 and Q793 had leukemogenic potential by delayed receptor internalization due to loss of internalization motifs (aa 772–778 and 779–792), whereas truncation mutation between Q793 and Q823 reduced receptor degradation due to loss of de-phosphorylation domain (aa N818-F836). Of interest, CSF3R type II mutations exhibited sensitivity to dasatinib treatment [12, 49].

In a patient with CMML, we found an uncommon missense variant in the cytoplasmic tail of CSF3R (p.M696T) defined as likely pathogenic. This mutation was previously found in 1 patient with CNL among 12 WHO-defined CNL cases [7]. In 19 consecutive patients with CNL, cases presenting with CSF3R T618I variant showed higher lymphocyte counts, lower hemoglobin value and platelet count, and worse clinical outcome compared to other CSF3R mutations i.e., M696T and T640N, suggesting that the type of CSF3R mutations could identify phenotypically and prognostically different subsets in CNL [50]. Some CSF3R variants in the cytoplasmic tail of the receptor, including M696T, P706C, P733T, E808K, and R698C mutations, were reported to lack the transformative capacity in the Ba/F3 cytokine independent model. M696T variant was also observed in three CMML patients as a somatic mutation [35], and in a patient with Ph + acute lymphoblastic leukemia as a germline variant, in all cases marginally contributing to leukemic transformation [51]. We did not have access to germline DNA to confirm the somatic nature of the CSF3R M696T variant in our patient; however, differently from patient with CMML and T618I mutation we couldn’t observe in this case a myeloproliferative phenotype, suggesting a marginal effect of this missense variant of cytoplasmic tail on proliferation.

About one half of CSF3R mutations in AML patients were localized in the extracellular domain, all but 2 being missense variants. Most truncating or missense mutations in the extracellular domain of CSF3R were reported to be loss-of-function alterations associated with neutropenia [52, 53]. These extracellular domain mutations are commonly associated with SCN and chronic idiopathic neutropenia and generally act in a dominant negative manner. Two mutations localized in the extracellular domain mutations were truncating variants; the nonsense p.W202* variant was observed in one patient with AML and p.P146Argfs in one CMML. It remains to be defined if this type of mutations in the extracellular domain are associated with neutropenia in other myeloid neoplasia than SNC.

In conclusion, CSF3R mutations are observed in about 3% of AML patients and more frequently (7%) in CMML, being pathogenetic or likely pathogenetic in most cases. Different classes of mutations seem to account for distinct phenotypic features with truncating or frameshift variants (class II) in the cytoplasmic tail of CSF3R being associated with increased proliferative promptness, whereas proximal membrane variants (class I) showing association with boost to myeloid differentiation, but additional studies are necessary to confirm the biological effect of different CSF3R variants in the contest of specific myeloid neoplasia and defined co-mutational pattern. In our cohort, two out of 5 patients with de novo AML and CSF3R pathogenic variants in the cytoplasmic tail were found to be primary refractory to induction therapy, comprising one case with NPM1-mutated AML and ELN favorable risk. However, the clinical impact of these mutations remains unknown and larger series of myeloid neoplasia are necessary to define the effect of CSF3R mutations on clinical outcome in patients with AML and CMML and to assess the therapeutic potential of kinase inhibitors (in particular, ruxolitinib or dasatinib) targeting CSF3R mutations.

## Electronic supplementary material

Below is the link to the electronic supplementary material.

Supplementary Material 1

## Data Availability

No datasets were generated or analysed during the current study.

## Abbreviations

AML: Acute myeloid leukemia
CMML: Chronic myelomonocytic leukemia
MDS: Myelodysplastic syndrome
PV: Polycythemia vera
MF: Myelofibrosis
VAF: Variant allele frequency
ACMG: American College of Medical Genetics and Genomics
VUS: Variant of uncertain significance
P: Pathogenic
LP: Likely pathogenic
