# Supplementary material for: Distribution of different classes of CSF3R mutations and co-mutational pattern in 360 myeloid neoplasia
Source: Ann Hematol. 2025 Feb 5;104(1):263–74. doi: 10.1007/s00277-025-06232-1 (PMC11868254; doi:10.1007/s00277-025-06232-1)
Supplement: Supplementary file 1 — Supplementary Material 1 [file 277_2025_6232_MOESM1_ESM.docx]

**Supplementary Information**

**Distribution of different classes of CSF3R mutations and co-mutational pattern in 360 myeloid neoplasia**

R. Maffei^1^, A. Paolini^1^, B. Conte^1,2^, G. Riva^1^, V. Nasillo^1^, F. Cretì^2^, S. Martinelli^1^, F. Giacobbi^1^, G. Corradini^1^, F. Pilato^1^, D. Bernabei^1^, C. Lancellotti^3^, G. Debbia^2^, M. Morselli^2^, L. Potenza^2^, D. Giusti^2^, E. Colaci^2^, F. Bettelli^2^, P. Bresciani^2^, A. Cuoghi^2^, A. Gilioli^2^, A. Messerotti^2^, V. Pioli^2^, M. Maccaferri^2^, G. Leonardi^2^, R. Manfredini^4,5^, R. Marasca^2^, A. Eccher^3^, M. Luppi^2*^, F. Forghieri^2*^, A. Candoni^2*^, E. Tagliafico^2,1*^

1. Department of Laboratory Medicine and Pathology, Diagnostic Hematology and Clinical Genomics, Azienda Ospedaliero-Universitaria, Policlinico, and AUSL Modena

2. Hematology Unit, Department of Medical and Surgical Sciences, University of Modena and Reggio Emilia, Azienda Ospedaliero-Universitaria, Policlinico, Modena

3. Pathology Unit, Department of Medical and Surgical Sciences, University of Modena and Reggio Emilia, Azienda Ospedaliero-Universitaria, Policlinico, Modena

4. Interdepartmental Centre for Stem Cells and Regenerative Medicine, University of Modena and Reggio Emilia; Modena, Italy

5. Department of Biomedical, Metabolic and Neural Sciences, University of Modena and Reggio Emilia, Modena

** Equally contributing to this work*

Corresponding authors:

Dr. Maffei Rossana, PhD, Biologist
SSD of Diagnostic Hematology and Clinical Genomics

Laboratory of Molecular Hematology

AOU of Modena- Policlinico

Padiglione Beccaria
Via Del Pozzo 71, 41124 Modena
tel. +39 059 4222715

e-mail: [rossana.maffei@unimore.it](mailto:rossana.maffei@unimore.it)

**Supplementary Table 1. Mutational profile of AML patients harboring CSF3R mutated gene**

| *Patient* | *Gene* | *Reference* | *Exon* | *Variant (nucleotide)* | *Variant (protein)* | *Mutation type* | *Allelic frequency VAF (%)* | *Pathogenicity* |
| --- | --- | --- | --- | --- | --- | --- | --- | --- |
| Case#1 | CSF3R | NM_000760 | 17 | c.2346dup | p.(Ser783Glnfs*6) | frameshift | 41.8 | P |
|  | CSF3R | NM_000760 | 14 | c.1853C>T | p.(Thr618Ile) | missense | 44.0 | P |
|  | DNMT3A | NM_022552 | 17 | c.1958T>A | p.(Leu653*) | nonsense | 47.8 | LP |
|  | DNMT3A | NM_022552 | 18 | c.2146G>A | p.(Val716Ile) | missense | 46.8 | LP |
| Case#2 | NPM1 | NM_002520 | 11 | c.863_864insCCAG | p.(Trp288Cysfs*?) | frameshift | 39.5 | P |
|  | CSF3R | NM_000760 | 17 | c.2361T>G | p.(Tyr787*) | nonsense | 38.9 | LP |
|  | DNMT3A | NM_022552 | 23 | c.2644C>T | p.(Arg882Cys) | missense | 43.4 | P |
| Case#3 | CSF3R | NM_000760 | 11 | c.1474+1G>A | p.(?) | splice_donor_+1 | 22.5 | LP |
|  | EZH2 | NM_004456 | 20 | c.2226_2227insACA | p.(Val742_Gly743insThr) | inframe_3 | 44.6 | VUS |
| Case#4 | ASXL1 | NM_015338 | 13 | c.1934dupG | p.(Gly646Trpfs*12) | frameshift | 28.9 | P |
|  | CSF3R | NM_000760 | 15 | c.1931_1935delinsAGTTCCACAGA | p.(Gly644_Thr645delinsGluPheHisArg) | inframe_6 | 12.8 | LP |
|  | KIT | NM_000222 | 17 | c.2458G>T | p.(Asp820Tyr) | missenso | 10.5 | P |
|  | NRAS | NM_002524 | 2 | c.35G>T | p.(Gly12Val) | missenso | 9.9 | P |
| Case#5 DIAGNOSIS | CEBPA | NM_004364 | 1 | c.198_201dup | p.(Ile68Leufs*41) | frameshift | 80.2 | P |
|  | ZRSR2 | NM_005089 | 7 | c.515G>T | p.(Cys172Phe) | missenso | 76.7 | LP |
|  | TET2 | NM_017628 | 3 | c.3398dupG | p.(Cys1133Trpfs*6) | frameshift | 40.8 | LP |
|  | DNMT3A | NM_022552 | 22 | c.2500A>T | p.(Thr834Ser) | missenso | 40.4 | LP |
| Case#5 RELAPSE | CSF3R | NM_000760 | 14 | c.1853C>T | p.(Thr618Ile) | missenso | 38.6 | P |
|  | TET2 | NM_001127208 | 11 | c.5734C>G | p.(His1912Asp) | missenso | 36.9 | LP |
|  | CEBPA | NM_004364 | 1 | c.198_201dup, | p.(Ile68Leufs*41) | frameshift | 79.0 | P |
|  | ZRSR2 | NM_005089 | 7 | c.515G>T | p.(Cys172Phe) | missenso | 80.2 | LP |
|  | TET2 | NM_017628 | 3 | c.3398dupG | p.(Cys1133Trpfs*6) | frameshift | 40.7 | LP |
|  | DNMT3A | NM_022552 | 22 | c.2500A>T | p.(Thr834Ser) | missenso | 41.1 | LP |
| Case#6 | ASXL1 | NM_015338 | 13 | c.1934dup | p.(Gly646Trpfs*12) | frameshift | 4.5 | P |
|  | SF3B1 | NM_012433 | 14 | c.1997A>C | p.(Lys666Thr) | missense | 6.5 | P |
|  | CSF3R | NM_000760 | 6 | c.606G>A | p.(Trp202*) | nonsense | 6.8 | LP |
|  | DNMT3A | NM_022552 | 16 | c.1904G>A | p.(Arg635Gln) | missense | 22.2 | P |
| Case#7 | PTPN11 | NM_002834 | 3 | c.205G>A | p.(Glu69Lys) | missense | 45.2 | P |
|  | TET2 | NM_017628 | 3 | c.1630C>T | p.(Arg544*) | nonsense | 45.4 | LP |
|  | DNMT3A | NM_022552 | 20 | c.2374C>T | p.(Arg792Cys) | missense | 86.3 | LP |
|  | TET2 | NM_001127208 | 9 | c.4172G>A | p.(Gly1391Asp) | missense | 43.4 | LP |
|  | CSF3R | NM_000760 | 9 | c.1028G>A | p.(Arg343Gln) | missense | 49.5 | VUS |
| Case#8 | TP53 | NM_000546 | 8 | c.799C>T | p.(Arg267Trp) | missense | 46.5 | P |
|  | CALR | NM_004343 | 9 | c.1099_1150del | p.(Leu367Thrfs*?) | frameshift | 39.6 | P |
|  | NRAS | NM_002524 | 2 | c.38G>A | p.(Gly13Asp) | missense | 28.9 | P |
|  | TET2 | NM_017628 | 3 | c.3410G>A | p.(Gly1137Asp) | missense | 67.1 | LP |
|  | CSF3R | NM_000760 | 10 | c.1249T>A | p.(Ser417Thr) | missense | 49.5 | VUS |
| Case#9 | FLT3 | NM_004119 | 20 | c.2503G>C | p.(Asp835His) | missense | 9.0 | P |
|  | NPM1 | NM_002520 | 11 | c.863_864insTAAG | p.(Trp288Cysfs*) | frameshift | 8.6 | P |
|  | CSF3R | NM_000760 | 13 | c.1616G>A | p.(Gly539Asp) | missense | 47.5 | VUS |
| Case#10 | IDH2 | NM_002168 | 4 | c.419G>A | p.(Arg140Gln) | missense | 50.1 | P |
|  | ASXL1 | NM_015338 | 13 | c.1934dupG | p.(Gly646Trpfs*12) | frameshift | 44.7 | P |
|  | CSF3R | NM_000760 | 7 | c.815C>T | p.(Pro272Leu) | missense | 50.5 | VUS |
|  | SRSF2 | NM_003016 | 1 | c.284C>T | p.(Pro95Leu) | missense | 48.4 | P |
| Case#11 | TP53 | NM_000546 | 3 | c.96+1G>A | p.(?) | splice_donor_+1 | 37.6 | P |
|  | CSF3R | NM_000760 | 11 | c.1456A>G | p.(Thr486Ala) | missense | 46.5 | VUS |
| Case#12 | IDH2 | NM_002168 | 4 | c.419G>A | p.(Arg140Gln) | missenso | 47.3 | P |
|  | CEBPA | NM_004364 | 1 | c.987dupA | p.(Gln330Thrfs*) | frameshift | 6.1 | P |
|  | RUNX1 | NM_001754 | 8 | c.961dupC | p.(Leu321Profs*) | frameshift | 47.0 | P |
|  | CSF3R | NM_000760 | 7 | c.722C>T | p.(Ala241Val) | missenso | 48.7 | VUS |
|  | DNMT3A | NM_022552 | 23 | c.2645G>A | p.(Arg882His) | missenso | 47.0 | P |
|  | SRSF2 | NM_003016 | 1 | c.284C>A | p.(Pro95His) | missenso | 47.2 | P |
| Case#13 | CSF3R | NM_172313 | 18 | c.2264G>C | p.(Arg755Pro) | missenso | 49 | VUS |

*Abbreviations: P, pathogenic variant; LP, likely pathogenic variant; VUS, variant of uncertain significance*

**Supplementary Table 2. Mutational profile of patients with myeloid neoplasia other than AML, harboring CSF3R mutated gene**

| *Patient* | *Gene* | *Reference* | *Exon* | *Variant (nucleotide)* | *Variant (protein)* | *Mutation type* | *Allelic frequency, VAF (%)* | *Pathogenicity* |
| --- | --- | --- | --- | --- | --- | --- | --- | --- |
| Case#14  CMML | DNMT3A | NM_022552 | 19 | c.2207G>T | p.(Arg736Leu) | missense | 48.0 | P |
|  | SRSF2 | NM_003016 | 1 | c.284C>T | p.(Pro95Leu) | missense | 47.3 | P |
|  | RUNX1 | NM_001754 | 8 | c.847C>T | p.(Gln283*) | nonsense | 46.3 | P |
|  | SF3B1 | NM_012433 | 14 | c.1998G>C | p.(Lys666Asn) | missense | 46.2 | P |
|  | CSF3R | NM_000760 | 14 | c.1853C>T | p.(Thr618Ile) | missense | 12.1 | P |
| Case#15  CMML | CSF3R | NM_000760 | 5 | c.437_438del | p.(Pro146Argfs*3) | frameshift | 49.7 | LP |
|  | CSF3R | NM_156039 | 17 | c.2087T>C | p.(Met696Thr) | missense | 49.7 | LP |
| Case#16  CNL | ASXL1 | NM_015338 | 13 | c.1900_1922del | p.(Glu635Argfs*15) | frameshift | 19.9 | P |
|  | CSF3R | NM_000760 | 17 | c.2372G>A | p.(Trp791*) | nonsense | 41.3 | P |
|  | CSF3R | NM_000760 | 14 | c.1853C>T | p.(Thr618Ile) | missense | 40.9 | P |
| Case#17  hypereosinophilia | CSF3R | NM_000760 | 11 | c.1474+1G>C | p.(?) | splice_donor_+1 | 49.2 | LP |
| Case#18  SMF | MPL | NM_005373 | 10 | c.1514G>A | p.(Ser505Asn) | missense | 45.4 | P |
|  | TET2 | NM_017628 | 3 | c.317del | p.(Leu106Profs*7) | frameshift | 42.9 | LP |
|  | CSF3R | NM_000760 | 17 | c.2242G>A | p.(Asp748Asn) | missense | 49.9 | VUS |
| Case#19  PMF | JAK2 | NM_004972 | 14 | c.1849G>T | p.(Val617Phe) | missense | 46.0 | P |
|  | SF3B1 | NM_012433 | 14 | c.1998G>T | p.(Lys666Asn) | missense | 44.7 | P |
|  | CSF3R | NM_000760 | 5 | c.402C>A | p.(Asn134Lys) | missense | 46.4 | VUS |
|  | SRSF2 | NM_003016 | 1 | c.284C>A | p.(Pro95His) | missense | 48.5 | P |
| Case#20  MDS | NRAS | NM_002524 | 2 | c.38G>A | p.(Gly13Asp) | missense | 7.6 | P |
|  | TET2 | NM_001127208 | 11 | c.4748C>A | p.(Ser1583*) | nonsense | 13.1 | P |
|  | CSF3R | NM_000760 | 12 | c.1540C>T | p.(Pro514Ser) | missense | 48.2 | VUS |

*Abbbreviations: P, pathogenic variant; LP, likely pathogenic variant; VUS, variant of uncertain significance*

**Supplementary table 3. Clinical characteristics of AML patients harboring pathogenic or likely pathogenic variants in CSF3R gene**

| *No* | *Diagnosis* | *Age-sex* | *Leukocytosis k/μl* | *neutrophils (%)* | *Blast BM (%)* | *BM dysplasia* | *BM fibrosis* | *Hb (g/dL)* | *PTL k/μl* | *first-line therapy* | *response* | *follow-up* |
| --- | --- | --- | --- | --- | --- | --- | --- | --- | --- | --- | --- | --- |
| AML#1 | De Novo AML | F-51 | 100.0 | 5% | 60% | yes | MF-0 | 12.8 | 30 | CPX-351 | primary refractory/savage therapy | alive-6 months |
| AML#2 | De Novo AML | M-57 | 13.7 | 22% | 60% | no | MF-1 | 5.9 | 181 | "7+3" scheme plus GO | primary refractory/HSCT | alive-28 months |
| AML#3 | De Novo AML | M-46 | 37.6 | 19% | 70% | no | NA | 4.8 | 16 | "7+3" scheme | complete response | dead-NA |
| AML#4 | De Novo AML | M-70 | 2.8 | 17% | 30% | no | NA | 10.3 | 99 | 5-aza plus venetoclax | complete response | relapsed/dead-10 months |
| AML#5 | Relapsed AML | M-78 | 5.5 | 67% | 7% | no | no | 16.8 | 129 | low-dose cytarabine | refractory | dead-20 months |
| AML#6 | MDS/AML | M-81 | 2.3 | 68% | 12% | yes | no | 9.8 | 310 | erythropoietin | stable disease | alive-16 months |

NA= Not available

**Supplementary table 4. Clinical characteristics of AML patients harboring variants of uncertain significance in CSF3R gene**

| *No* | *Diagnosis* | *Age-sex* | *Leukocytosis k/μl* | *neutrophils (%)* | *Blast BM (%)* | *BM dysplasia* | *BM fibrosis* | *Hb (g/dL)* | *PTL k/μl* | *first-line therapy* | *response* | *follow-up* |
| --- | --- | --- | --- | --- | --- | --- | --- | --- | --- | --- | --- | --- |
| AML#7 | De Novo AML | M-69 | 15.2 | 10% | 90% | yes | MF-0 | 11.2 | 52 | 5-aza plus venetoclax | complete response | alive-16 months |
| AML#8 | De Novo AML | F-72 | 29.2 | 69% | >30% | no | MF-3 | 9.2 | 128 | 5-aza plus venetoclax | NA | NA |
| AML#9 | De Novo AML | F-29 | 11.2 | 83% | 70% | NA | NA | 11.0 | 291 | "7+3" plus midostaurine | complete response/relapsed/HSCT | dead-16 months |
| AML#10 | De Novo AML | M-75 | 2.8 | 26% | 21% | yes | MF-0/1 | 12.6 | 78 | 5-aza plus venetoclax | stable disease | dead-6 months |
| AML#11 | De Novo AML | M-82 | 1.9 | 12% | 20% | yes | MF-0/1 | 7.5 | 24 | 5-aza | partial response | dead-11 months |
| AML#12 | De Novo AML | F-81 | 26.3 | 15% | NA | NA | NA | 4.6 | 9 | hydroxyurea | progressive disease | dead-4 months |
| AML#13 | myeloid sarcoma | M-22 | 6.0 | 59% | 30% | no | no | 14.7 | 190 | cytarabine, daunorubicine, etoposide | complete response/relapsed/FLAG-Ida | dead-8 months |

NA= Not available

**Supplementary table 5. Clinical characteristics of patients with myeloid neoplasia other than AML harboring variants in CSF3R gene**

| No | Diagnosis | *Age-sex* | *Leukocytosis k/μl* | *neutrophils (%)* | *Blast BM (%)* | *BM dysplasia* | *BM fibrosis* | *Hb (g/dL)* | *PTL k/μl* | *first-line therapy* | *response* | *follow-up* |
| --- | --- | --- | --- | --- | --- | --- | --- | --- | --- | --- | --- | --- |
| 14 | CMML-II | M-77 | 25.9 | 77% | 15% | yes | MF-0 | 9.2 | 45 | erythropoietin | progression (AML) | dead-10 months |
| 15 | CMML-I | M-81 | 5.9 | 56% | 3% | no | MF-0 | 12.3 | 82 | observation | - | alive-13 months |
| 16 | CNL | F-73 | 22.1 | 78% | <5% | no | MF-1 | 14.7 | 295 | observation | - | alive-106 months |
| 17 | Hypereosinophilia | M-24 | 19.4 | 29% | NA | NA | NA | 14.9 | 260 | observation | - | alive-26 months |
| 18 | secondary MF (Post-PV) | M-60 | 3.8 | 63% | <5% | no | MF-1 | 13.2 | 628 | observation | - | alive-27 months |
| 19 | MF | M-67 | 17.8 | 86% | <5% | yes | MF-1 | 6.9 | 401 | NA | NA | NA |
| 20 | MDS | M-69 | NA | NA | <5% | no | no | NA | NA | NA | NA | NA |

NA= Not available
